# IL-6 Stabilizes Twist and Enhances Tumor Cell Motility in Head and Neck Cancer Cells through Activation of Casein Kinase 2

**DOI:** 10.1371/journal.pone.0019412

**Published:** 2011-04-29

**Authors:** Ying-Wen Su, Tong-Xin Xie, Daisuke Sano, Jeffrey N. Myers

**Affiliations:** 1 Program in Cancer Biology, The University of Texas Graduate School of Biomedical Sciences, Houston, Texas, United States of America; 2 Department of Head and Neck Surgery, The University of Texas MD Anderson Cancer Center, Houston, Texas, United States of America; Karolinska Institutet, Sweden

## Abstract

**Background:**

Squamous cell carcinoma of the head and neck (SCCHN) is the seventh most common cancer worldwide. Unfortunately, the survival of patients with SCCHN has not improved in the last 40 years, and thus new targets for therapy are needed. Recently, elevations in serum level of interleukin 6 (IL-6) and expression of Twist in tumor samples were found to be associated with poor clinical outcomes in multiple types of cancer, including SCCHN. Although Twist has been proposed as a master regulator of epithelial-mesenchymal transition and metastasis in cancers, the mechanisms by which Twist levels are regulated post-translationally are not completely understood. Tumor progression is characterized by the involvement of cytokines and growth factors and Twist induction has been connected with a number of these signaling pathways including IL-6. Since many of the effects of IL-6 are mediated through activation of protein phosphorylation cascades, this implies that Twist expression must be under a tight control at the post-translational level in order to respond in a timely manner to external stimuli.

**Methodology/Principal Findings:**

Our data show that IL-6 increases Twist expression via a transcription-independent mechanism in many SCCHN cell lines. Further investigation revealed that IL-6 stabilizes Twist in SCCHN cell lines through casein kinase 2 (CK2) phosphorylation of Twist residues S18 and S20, and that this phosphorylation inhibits degradation of Twist. Twist phosphorylation not only increases its stability but also enhances cell motility. Thus, post-translational modulation of Twist contributes to its tumor-promoting properties.

**Conclusions/Significance:**

Our study shows Twist expression can be regulated at the post-translational level through phosphorylation by CK2, which increases Twist stability in response to IL-6 stimulation. Our findings not only provide novel mechanistic insights into post-translational regulation of Twist but also suggest that CK2 may be a viable therapeutic target in SCCHN.

## Introduction

Squamous cell carcinoma of the head and neck (SCCHN) is the seventh most common cancer worldwide [1]. Despite improvements in surgical and radiation therapy techniques, the 5-year survival rate has not improved significantly over the past several decades and remains at 50–55%. Although local recurrence and neck lymph node metastases account for most of the deaths from this disease, only 10–20% of patients benefit from the integration of systemic chemotherapeutic therapy, with marginally improved survival and considerable toxic effects [2], [3]. Therefore, new targets for therapy are needed.

Recently, overexpression of Twist in clinical tumor specimens was found to be correlated with metastasis and poor prognosis in patients with SCCHN as well as other cancers [4]–[7]. Twist is a highly conserved basic-helix-loop-helix transcription factor that plays an important role in facilitating cell movement in the development of embryos. In cancer cells, Twist is regarded as an oncogene, as its elevated expression promotes disease progression and metastasis by inducing the epithelial-mesenchymal transition (EMT) [8].

### Despite its importance in tumor progression, post-transcriptional regulation of Twist is not well understood

A comparative analysis of Twist mRNA and Twist protein expression in mouse embryos showed abundant Twist RNA expression in presomitic mesoderm, epithelial somites, and anterior mesoderm, but no Twist protein could be found in those tissues [9]. The discrepancy was also noted during mouse embryo development, as Twist RNA reaches its highest level at 7.0 days post coitum while no Twist protein could be found prior to 8.25 days post coitum. The lack of concordance between Twist mRNA expression and Twist protein expression indicates that Twist expression is controlled at the post-transcriptional level [9]. Post-transcriptional modification of transcription factors, including phosphorylation and ubiquitination, has been shown to be important for their function, as this provides a mechanism by which the cell can rapidly initiate transcriptional programs in response to external stimuli. For example, it has been reported that Twist can be degraded through the ubiquitin/proteasome degradation pathway, as treatment with a proteasome inhibitor inhibits degradation of Twist [10]. There is also evidence that the function of Twist can be modulated by phosphorylation [11], [12]. Because phosphorylation is often involved in the regulation of a protein's ubiquitin/proteasome-dependent degradation [13], we hypothesized that phosphorylation of Twist increases its stability by increasing its relative expression level.

SCCHN tumorigenesis and progression are known to be influenced by multiple growth factors and cytokine signaling factors, including interleukin 6 (IL-6) [14]–[17]. In SCCHN patients, elevated serum IL-6 level correlates with poor survival and unfavorable clinical outcome [14], [15], [18]. IL-6, produced either by infiltrating immune cells or tumor cells, not only provides survival signals to cancer cells but also facilitates motility of cancer cells through the EMT [19], [20]. The traditional IL-6 signaling pathway is through binding with sIL-6 receptor (gp80), which induces dimerization of gp130 and subsequent activation of either Janus kinases (JAK)/STAT3 in a transcription-dependent manner, or the Ras-MAPK and PI3K/Akt pathway [21]. In addition to canonical pathways, casein kinase 2 (CK2) has also recently been reported downstream of IL-6 signaling in cancer [22].

CK2 is a highly conserved and ubiquitously expressed serine/threonine kinase, which consists of two catalytic (α or α') and two β regulatory subunits [23]. The importance of CK2 subunits can be demonstrated by genetic studies showing that mice lacking CK2α or CK2β are embryonic lethal while CK2 α' knockout mice had defects only in spermatogenesis [24]–[26]. CK2 has recently come to be regarded as a “master kinase” since it controls the activity of many other kinases and is involved in many important cellular processes [27]. For example, CK2 controls the stability of IκBα and PML tumor suppressor through phosphorylation and modification of their ubiquitin/proteasome degradation [28], [29]. CK2 has been reported to be overexpressed and to correlate with poor survival in many tumor types, including SCCHN [30]–[32]. Traditionally, CK2 is regarded as a constitutively active protein kinase, but several studies have shown that CK2 can respond to many growth factor stimuli, including IL-6 [22], [33], although the mechanisms of its activation remain largely unclear [34].

It has been reported that STAT3, the major downstream signal of the IL-6 pathway, can transcriptionally activate the expression of Twist [35], [36]. Our preliminary data showed, however, that Twist protein expression was increased by IL-6 before the upregulation of Twist mRNA in multiple aggressive SCCHN cell lines, suggesting a transcription-independent regulation of Twist by IL-6. Since many of the effects of IL-6 are mediated through activation of protein phosphorylation cascades [21], and substrate phosphorylation is often involved in regulation of a protein's ubiquitin/proteasome−dependent degradation [37], we postulated that Twist expression is regulated by IL-6−activated phosphorylation.

In this study, we demonstrate that treatment of SCCHN cell lines with IL-6 leads to stabilization of the Twist protein. Further investigation showed that IL-6 stimulates Twist phosphorylation through activation of the CK2 serine/threonine protein kinase. Our findings not only provide a novel mechanistic insight that Twist is activated through phosphorylation by the CK2 kinase but also have significant implications for the prognostication and treatment of head and neck cancer.

## Results

### Twist expression is upregulated shortly after IL-6 treatment

**Figure 1:**
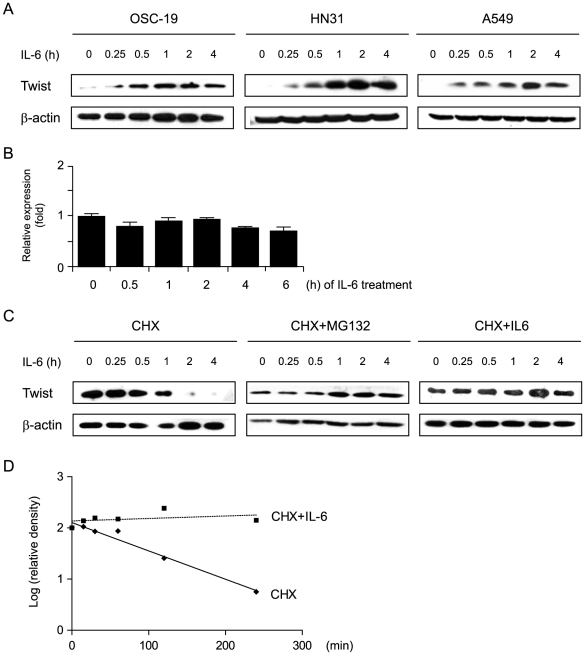
IL-6 upregulates Twist expression by inhibiting its degradation. (A) Twist protein expression was induced shortly after IL-6 treatment in SCCHN cells (OSC-19, HN31) and lung cancer cells (A549). After serum starvation overnight, cells were subjected to IL-6 treatment (20 ng/ml for 0–4 h), and Twist expression in cell lysates was analyzed by western blot. β-actin was used as a loading control. (B) Twist mRNA expression as quantified by real-time RT-PCR remained mostly unchanged throughout IL-6 treatment (0–6 hours). Values were normalized to the expression levels of the housekeeping gene *GAPDH*, and are expressed as the mean fold change from basal level ± s.e.m. For each time point, two to four replicates were performed. All experiments were done in duplicate for each cell line. Data from a representative experiment with OSC-19 SCCHN cells are shown. (C) Twist degradation was inhibited by either MG132 or IL-6. After OSC-19 SCCHN cells were treated with CHX (100 µM), CHX plus the proteasome inhibitor MG132 (10 µM), or CHX plus IL-6 (20 ng/ml) for 0–4 h, Twist expression was determined by western blot. (D) The pixels in each band in (C) were measured by Image J and normalized so that the number of pixels at time 0 was 100%. The data are plotted as the log_10_ of the relative pixel density (y-axis) versus the time (min). The half-life was determined from the log of 50% relative pixel density. The half-life for Twist was 1.2 h in the presence of CHX alone and >4 h in the presence of CHX+IL-6.

Most SCCHN and non-small cell lung cancer (NSCLC) cell lines secrete IL-6 and express receptors for IL-6 (Table S1 and Figure S1) [38]. To study the impact of IL-6 on these cell lines, Twist expression was examined along the time course of treatment. Western blots from representative cell lines are shown in Figure 1A. Twist expression was induced in these cells within 15 min after treatment with IL-6. In contrast, Twist mRNA levels remained unchanged over a similar treatment period (Figure 1B). These data indicate that Twist expression is regulated by IL-6 at the post-transcriptional level.

This observation that IL-6 upregulates Twist expression post-transcriptionally led us to investigate whether degradation of Twist was in turn modulated by IL-6 treatment. The degradation rates of Twist in SCCHN cells were examined by treating with protein synthesis inhibitor cycloheximide (CHX) alone, CHX plus the proteasome inhibitor MG132, or CHX plus IL-6. The amount of protein at each time point was determined by western blot and quantified by densitometry. As shown in Figures 1C and 1D, endogenous Twist had a 1.2-h half-life, but when either IL-6 or MG132 was added, less Twist was degraded and it did not reach its half-life in the 4-h treatment period. The data are consistent with an earlier study showing that Twist protein is degraded through the proteasome degradation system [10] and indicate that the Twist protein is stabilized post-translationally through inhibition of its degradation by IL-6.

### Twist is phosphorylated in response to IL-6 treatment, and CK2 is found in the pathway between IL-6 and Twist

**Figure 2:**
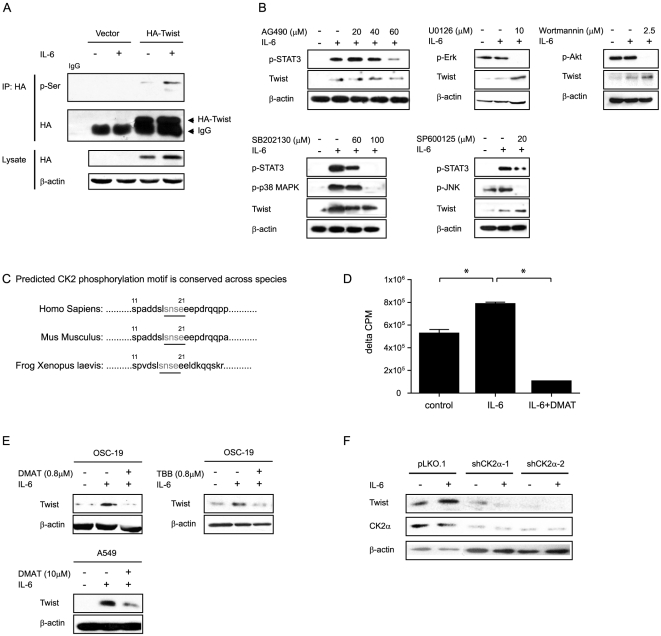
CK2 is in the pathway of IL-6 signaling. (A) Twist was phosphorylated in response to IL-6 treatment. OSC-19 SCCHN cells were transfected with either the HA-Twist plasmid or control vector for 48 h, then treated with IL-6 (20 ng/ml) for 30 min; treated and untreated cell lysates were immunoprecipitated with HA antibody and analyzed by western blot using a nonspecific phosphoserine antibody. (B) Inhibitors to known downstream pathways of IL-6 were unable to inhibit Twist upregulation by IL-6. OSC-19 SCCHN cells were pretreated with the indicated kinase inhibitors or dimethyl sulfoxide (DMSO; control) for 1 h, then were treated with IL-6 (20 ng/ml) for 30 min. Twist expression was determined by western blot. (C) The CK2 phosphorylation site (SNSE) in Twist, predicted by use of http://scansite.mit.edu/motifscan_seq.phtml, is conserved across species. (D) CK2 activity was increased by IL-6 and inhibited by CK2 inhibitor DMAT in SCCHN cells. After serum starvation overnight, lysates of HN31 cells treated with PBS (control), IL-6 (20 ng/ml, 30 min), or IL-6 plus DMAT (10 µM) were collected. Endogenous CK2 activity in the cell lysates (5 µg) was measured using a synthetic peptide CK2 substrate (RRRADDSDDDDD; 0.1 mM) and γ-^32^P-ATP as a phosphate donor as previously described [49]. The y-axis represents the count per minute (CPM) of the radioactivity after normalizing to the no-substrate control. * *P*<0.05 by the Student *t*-test. (E) IL-6−induced Twist expression was inhibited by CK2 inhibitors. Twist expression was measured after treatment with IL-6 (30 min) in lysates of OSC-19 SCCHN or A549 lung cancer cells previously incubated in CK2 inhibitors DMAT or TBB (1 h). Twist expression was inhibited at doses as low as 0.8 µM and was not restricted to a specific cell line. (F) Knockdown of the catalytic subunit of CK2 (CK2α) by shRNA inhibited Twist protein expression. Knockdown of CK2α in OSC-19 SCCHN cells for 24 h significantly reduced Twist expression and blocked its induction by IL-6 treatment (20 ng/ml, 30 min).

Because IL-6 has been shown to activate multiple cell signaling pathways through activation of protein kinase cascades, we next examined whether Twist is phosphorylated in response to IL-6 treatment. Cells transfected with a plasmid encoding a hemagglutinin (HA)-Twist fusion protein were treated with IL-6; subsequent immunoprecipitation with an HA antibody and western blotting using a nonspecific phosphoserine antibody revealed increased phosphorylation of serine/threonine residues in Twist (Figure 2A), confirming that Twist is phosphorylated in response to IL-6 stimulation.

To identify the kinase responsible for the IL-6−induced phosphorylation of Twist, we used a chemical inhibitor screening strategy to determine whether the IL-6−mediated Twist upregulation can be blocked by kinase inhibitors known to be downstream of the signaling pathway. Twist was upregulated by IL-6 despite application of inhibitors blocking JAK (AG490), PI-3K (Wortmannin), Erk (U0126), p38 MAPK (SB202130), or Jun *N*-terminal kinase (SP600125), indicating that Twist upregulation is independent of these pathways (Figure 2B). Since none of the known pathways that we tested appeared to be involved in mediating IL-6−induced Twist expression, we next scanned the amino acid sequence in the computational protein family prediction webserver http://scansite.mit.edu/motifscan_seq.phtml and identified a CK2 substrate consensus motif (SNSE) within Twist at residues 18 through 21 that is conserved across species (Figure 2C).

CK2 is a serine/threonine kinase; increasing recent studies indicate that it may play an important role in the progression of SCCHN [32], [39]. It was previously regarded as a constitutively active intracellular kinase, but several recent findings have shown that CK2 can be activated in response to external stimuli such as IL-6, although the underlying mechanisms for this activation remain unclear [22], [34]. Consistent with previous studies, CK2 activity in SCCHN cell lysates was upregulated by IL-6 and inhibited by CK2-specific inhibitor 2-dimethylamino-4, 5, 6, 7-tetrabromo-1H-benzimidazole (DMAT), as determined by a CK2 kinase activity assay that used a synthetic CK2 substrate peptide and γ-^32^P-ATP as a phosphate donor (Figure 2D).

To further demonstrate that CK2 is in the pathway between IL-6 and Twist, we next examined Twist expression in the presence of IL-6 and CK2 inhibitors DMAT or 4,5,6,7-tetrabromobenzotriazole (TBB). As shown in Figure 2E, IL-6−induced Twist expression was inhibited by either CK2 inhibitor and this inhibition was observed across different cell lines. The effects of CK2 on IL-6−induced Twist expression were further confirmed by knockdown of the catalytic subunit CK2α in SCCHN cell lines, in which both the basal levels of Twist expression and those after IL-6 treatment were reduced (Figure 2F). Taken together, these data further confirm the importance of CK2 to IL-6–induced stabilization of Twist.

### CK2 associates with, phosphorylates, and stabilizes Twist

**Figure 3:**
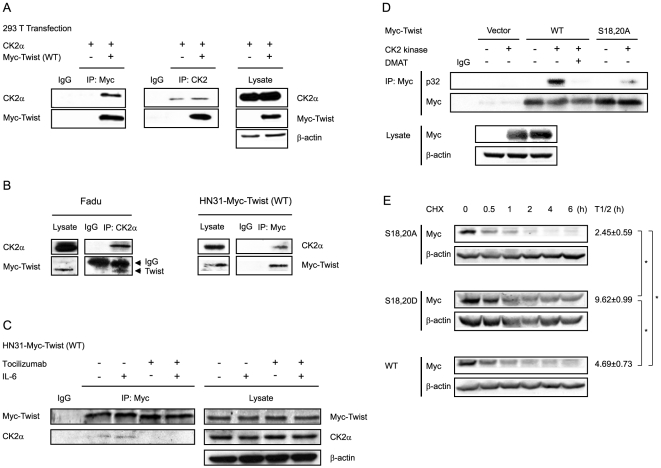
CK2α associates with, phosphorylates, and stabilizes Twist. (A)&(B) Bi-directional co-immunoprecipitation showed that CK2α is associated with Twist. (A) Co-immunoprecipitation was done in lysates from HEK 293T cells transfected with both WT Myc-Twist and CK2α. CK2α was co-immunoprecipitated with Myc-Twist and vice versa. (B) Cell lysates from FaDu (left) or HN31 cells, which stably express WT Myc-Twist (right), were immunoprecipitated with CK2α (in FaDu cells) or Myc antibody (in HN31 WT Myc-Twist cells) and subjected to western blot analysis. Endogenous Twist was co-immunoprecipitated with CK2α in FaDu cell lysates, and endogenous CK2α was found in the Myc-immunoprecipitates of HN31 Myc-WT Twist cell lysates. (C) Co-immunoprecipitated CK2α in the HN31 Myc-Twist immunoprecipitates was increased by IL-6 (20 ng/ml, 20 min) and inhibited by pretreatment with an antibody against IL-6 receptor, tocilizumab (50 ng/ml, 45 min). (D) CK2 phosphorylated Twist. An immunocomplex kinase assay was performed on Myc-immunoprecipitates from HEK 293T cell lysates transiently overexpressing WT Myc-Twist or Myc -S18,20A Twist. Recombinant CK2 kinase and γ-^32^P-ATP were added to the immunoprecipitates at 30°C for 15 min, and the reaction was stopped by addition of SDS sample buffer and heating at 95°C for 5–10 min. The samples were then subjected to electrophoresis and transferred onto a polyvinylidene fluoride membrane for autoradiography or western blot analysis. (E) The stability of Twist was affected by site of CK2 phosphorylation.[HN31 SCCHN cells stably expressing WT Myc-Twist, the CK2 hypophosphomimetic mutant Myc-S18,20A Twist, or the phosphorylation-mimetic mutant Myc-S18,20D Twist were treated with CHX (100 µM) for the indicated times. Myc-Twist expression in the cell lysates was determined by western blot. Data represent one of three independent experiments. The calculated half-lives (t1/2) from the triplicate experiments are expressed as mean ± s.e.m. * *P*<0.05 by the Student *t*-test.

We next examined whether CK2 and Twist are associated with one another by using co-immunoprecipitation experiments. First we used lysates prepared from HEK 293T cells transfected with both wild-type Myc-Twist and CK2α. As shown in Figure 3A, CK2α was detected in western blots of immunoprecipitates performed with Myc antibody and Myc-Twist was detected in the western blots of immunoprecipitates performed with CK2α antibody. Control immunoprecipitates using nonspecific IgG did not precipitate either protein. The data suggest that the proteins can interact with each other. To further examine the interaction between endogenous CK2α and Twist protein in SCCHN cells, the FaDu cell line was chosen because it expresses a high basal level of CK2 protein; HN31 SCCHN cells that stably express Myc-tagged Twist (HN31 Myc-Twist SCCHN cells) were established for immunoprecipitation purposes because of the poor performance of the commercially available Twist antibodies. As shown in Figure 3B, endogenous Twist in FaDu SCCHN cells was co-immunoprecipitated with an anti-CK2α antibody. In HN31 Myc-Twist SCCHN cells, which stably express Myc-tagged Twist, endogenous CK2α was also co-precipitated with the anti-Myc immunoprecipitates. These data support the hypothesis that CK2 can regulate Twist by showing that these two proteins physically associate with each other.

To examine whether the interaction between Twist and CK2α is IL-6 dependent, co-immunoprecipitation was repeated in Myc-Twist−stable cell line HN31 after brief treatment with IL-6 (20 min). Because HN31 expresses high levels of endogenous IL-6 (Table S1), a monoclonal antibody against the IL-6 receptor, tocilizumab, was used to examine their interaction under blockade of IL-6 signaling. As shown in Figure 3C, increased CK2α was co-immunoprecipitated with Myc-Twist after IL-6 treatment but was not found in the immunoprecipitates from cells pretreated with tocilizumab for 45 min.

To determine whether CK2 phosphorylates Twist at residues S18/S20, the putative phosphorylation site from our computational prediction, we performed an immunocomplex kinase assay in HEK 293T cells overexpressing either wild-type (WT) Myc-Twist or mutant Myc-Twist in which S18 and S20 are substituted with alanine (S18,20A Twist). Purified CK2 kinase phosphorylated WT Twist, but phosphorylation was abolished in the presence of the CK2 inhibitor DMAT and strongly reduced in mutated S18,20A Twist (Figure 3D).

To test whether the stability of Twist is affected by the site of phosphorylation, stable HN31 cells overexpressing WT Twist, S18,20A Twist, or a phosphorylation mimic in which S18 and S20 are mutated to aspartic acid (S18,20D Twist) were established. After treating the cells with CHX, the calculated half-lives for S18,20D Twist, S18,20A Twist, and WT Twist from the three experiments were 9.62±0.99 h for S18,20D Twist, 2.45±0.59 h for S18,20A Twist, and 4.69±0.73 h for WT Twist (Figure 3E). All *P* values for comparing means from any two groups with the Student *t*-test were less than 0.05. This supports the hypothesis that phosphorylation of Twist by CK2 enhances Twist stability. Taken together, these data indicate that CK2 associates with and phosphorylates Twist, and that phosphorylation at S18 and S20 stabilizes Twist.

### CK2 and Twist are involved in IL-6–promoted cell motility

**Figure 4:**
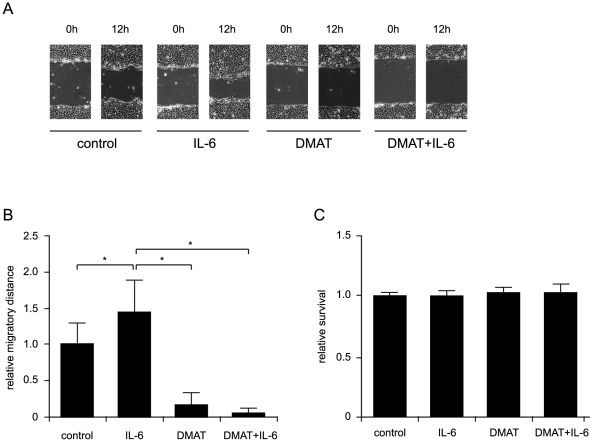
CK2 is involved in IL-6−promoted cell motility. (A) Images for SCCHN cell migration by wound-healing assay are shown. OSC-19 SCCHN cells were treated with PBS (control), IL-6 (20 ng/ml), DMAT (20 µM), or both IL-6 and DMAT in 2% medium as indicated. The confluent cell monolayers were wounded by scraping with a 200-µL pipette tip. Pictures of the scratches were acquired at 0 and 12 h. (B) Quantitative measurements of the relative migrated distance were analyzed with Image J. Data are expressed as mean ± s.e.m. of the relative migrated distance. * *P*<0.05 by the Student *t*-test. (C) Cell proliferation rate under each condition in (A) was measured by MTT assay for 12 h. Differences between groups have no statistical significance.

**Figure 5:**
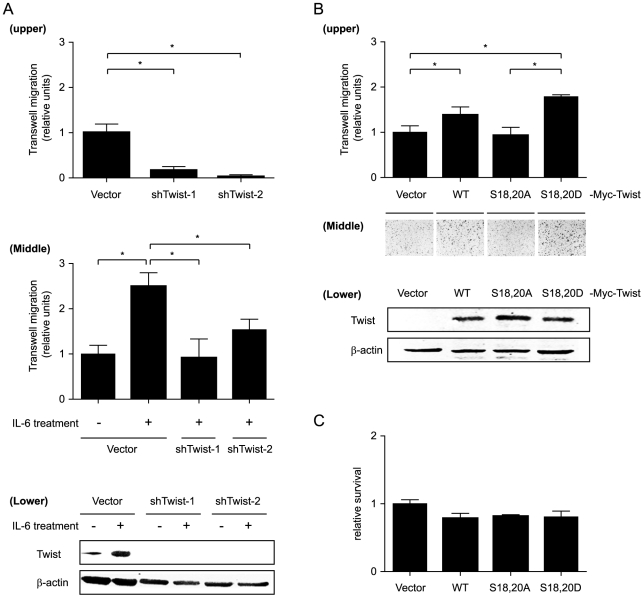
IL-6−induced cancer cell motility is, in part, acting through Twist. (A) Knockdown of Twist inhibited SCCHN cell migration in the Boyden chamber migration assay. OSC-19 SCCHN cells were first transfected with control vector or Twist shRNAs for 24 h in medium with or without IL-6 (20 ng/ml). Cells were then subjected to trypsinization, counted, and assayed for cell motility using a Boyd chamber migration assay after 20 h. Upper & middle panels: data are expressed as mean ± s.e.m. of the cell counts from four different lower power field microscopic views. * *P*<0.05 by the Student *t*-test. Lower panel: Twist expression for the corresponding experiments in the upper and middle panels was detected by western blot. β-actin was used as loading control. (B) Mutation of Twist altered migration of SCCHN cells. The motility of HN31 SCCHN cells stably expressing WT Myc-Twist or indicated mutant Myc-Twist protein was examined with the Boyden chamber migration assay after 20 h. Upper panel: data are expressed as mean ± s.e.m. of the cell counts from four different lower power field microscopic views. * *P*<0.05 by the Student *t*-test. Middle panel: representative images of the migrated cells from the corresponding transwell membranes at low power field magnification. Lower panel: western blots for the corresponding experiments in the upper panel of (B). β-actin was used as loading control. (C) Cell proliferation of each condition in (B) as measured by MTT assay for 20 h. Differences between groups have no statistical significance.

We next examined the effects of IL-6, CK2 inhibition, and Twist knockdown on SCCHN cell motility, as measured by wound-healing and Boyden-chamber migration assays. As shown in Figures 4A and 4B, the migration of OSC-19 SCCHN cells in a 12-h wound-healing scratch assay was promoted by IL-6 and suppressed by the CK2 inhibitor DMAT, while the relative proliferation rate in each group underwent no significant change (Figure 4C). Knockdown of Twist in OSC-19 cells profoundly suppressed cell motility, indicating its important role in cell migration (Figure 5A, upper panel). After treatment with IL-6 for 24 h, migration was increased, but this increase was reversed in the Twist knockdown groups (Figure 5A, middle panel). IL-6–promoted cell migration was inhibited by CK2 inhibitor as well as knockdown of Twist, suggesting that both CK2 and Twist are implicated in signal-induced migration. We next compared the motility of cell lines stably expressing WT or mutant Twist *in vitro*. Despite the background of high endogenous IL-6 secretion and existence of endogenous Twist in HN31 SCCHN cells, overexpression of WT, but not S18,20A, Twist promoted cell migration relative to the control (Figure 5B, upper panel; *P*<0.05). Furthermore, overexpression of S18,20D Twist further increased cell motility relative to that of cells expressing either WT or S18,20A Twist, suggesting that this mutation leads to enhanced cell motility.

## Discussion

This report demonstrates a novel post-translational phosphoregulation of Twist by IL-6 through activation of CK2 in SCCHN cells. This post-translational modification can stabilize Twist, allowing it to regulate cell motility, providing evidence of a new mechanism for Twist regulation in cancer cells.

Published reports have implicated both Twist and IL-6 in the development/progression of cancer, as their expressions are detectable in many epithelial tumors or patients' serum and are associated with unfavorable clinical outcomes [4], [16], [18]. Although it has been reported that IL-6 and its downstream signal mediator, STAT3, can increase Twist expression through transcription in breast cancer cell lines [20], [35], [36], this does not exclude the mechanism of post-translational modification of Twist expression. As shown in Figure 2B, Twist expression is induced shortly after IL-6 treatment despite inhibition of phospho-STAT3 by chemical inhibitor AG490 or SB202130 at higher doses, indicating that the Twist upregulation is STAT3 independent. As shown in Figures 1A and 1B, Twist protein expression markedly increased before changes in Twist mRNA expression in SCCHN cell lines. These findings do not contradict those of an earlier study by Lo *et al* [36], in which Twist protein was induced at 1 h after activation of the EGFR-phospho-STAT3 pathway while Twist mRNA levels increased only after 2 h of treatment. Although it was not discussed in the earlier work, the discrepancy between Twist mRNA and protein expression in our study indicates the existence of a post-transcriptional regulatory mechanism across different cell lines.

Our data demonstrate that Twist protein can be phosphorylated and stabilized by IL-6 in SCCHN cell lines, supporting the concept that phosphoregulation of transcriptional factors is often utilized by multiple cellular signaling pathways for timely response to external stimuli [37]. Although Twist phosphoregulation has been described in studies of Twist mutations in patients with Saethre-Chotzen syndrome, an autosomal dominant disorder of craniosynostosis [11], the role of phosphoregulation of Twist in cancer cells, to our knowledge, has been discussed in only a few studies [12], [40]. The CK2 phosphorylation site (S18 and S20) identified in this study is located within the NSEEE motif, one of five evolutionarily conserved domains in the Twist amino acid sequence, whose function is unclear [41]. Our finding may help improve understanding of this domain, since CK2 is connected to many growth factor or cytokine pathways, such as IL-6 and epidermal growth factor (EGF), in which Twist is also involved [20], [22], [33], [36].

Interestingly, these factors are also well known to influence tumorigenesis and progression of SCCHN [16]. We analyzed the post-transcriptional regulation of Twist in response to other SCCHN-relevant cytokines/growth factors and found that the function of stabilizing Twist is not limited to IL-6. Other important growth factors in SCCHN, such as EGF and vascular endothelial growth factor C, also stabilize Twist levels [Figure S2]. This suggests that a common mechanism may participate in regulation of Twist in SCCHN, and that it may be interesting to know the role of CK2 in these signaling pathways.

The mechanism through which CK2 is activated remains unclear [34]. Although it has been shown that CK2 activation is Erk-dependent in neuroblastoma cells [33], pharmacological inhibition of Erk with U0126 in our study did not block the IL-6−mediated increase in Twist expression (Figure 2B). This suggests that there may be cell mediators other than Erk that can activate CK2. Whether this is cell type specific needs to be investigated further.

Metastasis is the major threat to the health and survival of cancer patients and its management is challenging for health care professionals. Since Twist is regarded as a master regulator of EMT and metastasis for cancers [8], downregulation of Twist in cancer cells has been proposed as a promising therapeutic approach, as it not only sensitizes cells to chemotherapy and promotes cell apoptosis but also inhibits the cells' migratory and invasive abilities [33], [42]. Direct targeting of Twist remains elusive, however, as therapeutic small-interfering RNA delivery approaches have yet to be optimized. In this report, we show that the level of Twist expression can be modulated by using a pharmacological approach (i.e., a CK2 inhibitor), suggesting that CK2 could be a useful therapeutic target. Downregulation of CK2 has been shown to lead to reduced viability and motility in cultured SCCHN cells and to inhibit SCCHN tumor growth and metastasis *in vivo* [39], [43], [44]. Since many important regulatory cellular proteins are the targets of CK2 [27], and CK2 is oncogenic in transgenic mice [45], [46], it is not surprising that CK2 has previously attracted attention as a potential target for therapy. An oral CK2 inhibitor is now available and is being investigated clinically [47]; further preclinical and molecular studies are needed to define the therapeutic potential of CK2 in SCCHN.

## Materials and Methods

### Cell culture, reagents, and chemical compounds

FaDu, OSC-19, and HN31 are human SCCHN cell lines. The FaDu cell line was obtained from American Type Culture Collection (Manassas, VA, USA); OSC-19 was purchased from the Health Science Research Resources Bank in Japan (Sennan-shi, Osaka, Japan); and HN31 was obtained from Dr. John Ensley (Wayne State University, Detroit, MI, USA). A549 is a human lung cancer cell line and was obtained from Dr. Reuben Lotan (MD Anderson Cancer Center, Houston, TX, USA). All cells were tested and authenticated upon receipt at the Johns Hopkins Genetic Resources Core Facility (Baltimore, MD, USA). Unless stated otherwise, all cells were cultured in Dulbecco's modified Eagle's medium supplemented with 10% fetal bovine serum (FBS), L-glutamine, sodium pyruvate, nonessential amino acids, and a 2-fold vitamin solution (Life Technologies Inc, Grand Island, NY, USA). The A549 cell line was cultured in RPMI 1640 supplemented with 10% FBS. All cells were grown in a humidified incubator at 37°C in 5% carbon dioxide. The cultures were free of Mycoplasma species and maintained for no longer than 8 weeks after recovery from frozen stocks. Stable HN31 cell lines expressing WT Twist, S18,20A Twist, or S18,20D Twist were established in the presence of 500 µg/ml of G418 (Invitrogen, Carlsbad, CA, USA). Cycloheximide (CHX), MG132, 2-dimethylamino-4, 5, 6, 7-tetrabromo-1H-benzimidazole (DMAT), 4,5,6,7-tetrabromobenzotriazole (TBB), U0126, AG490, SB202130, and SP600125 were purchased from EMD Chemicals (Gibbstown, NJ, USA). Human recombinant IL-6 and CK2 kinase were purchased from Cell Signaling Technology (Danvers, MA, USA). The CK2 substrate peptide (RRRADDSDDDDD) was synthesized by GenScript (Piscataway, NJ, USA). Tocilizumab, a monoclonal antibody against human IL-6 receptor, was obtained from Chugai Pharmaceutical Co. Ltd. (Gotemba, Shizuoka, Japan).

### Plasmids, antibodies, transfection, and real-time reverse-transcription polymerase chain reaction

Short-hairpin (sh) RNAs specific for Twist were kind gifts of Dr. Lu-Hai Wang (Mount Sinai School of Medicine, New York, NY, USA). CK2α–CMV (clone id: 3908058, catalog number: MHS1010-9205500) and shRNAs specific for CK2α (NM_001895) (catalog numbers: RHS3979-9593435, RHS3979-98490747) were purchased from Open Biosystems (Huntsville, AL, USA). Myc-Twist (NM_000474) was cloned into pcDNA3.1+ using *Bam*H1/*Eco*R1 and standard polymerase chain reaction (PCR) procedures. Point mutations were introduced by PCR using the QuikChange site mutagenesis kit following the manufacturer's protocol (Stratagene, Cedar Creek, TX, USA). Antibodies recognizing Twist-1 and CK2α were purchased from Cell Signaling Technology. Transfection in HEK 293T was carried out by using Lipofectamine 2000 (Invitrogen) according to the manufacturer's instructions. Transfection in OSC-19 and HN31 was carried out with the Amexa Necleofector System (Lonza, Switzerland) programs V-020 and T-001, respectively. Relative quantities of mRNA expression were analyzed using real-time reverse-transcriptase PCR (Bio-Rad, Hercules, CA, USA) with the primers described previously (36;38). SYBR green fluorescence dye (Thermo-Scientific, Worcester, MA, USA) was used in the study.

### Western blot analysis, immunoprecipitation, and CK2 kinase activity assay

Western blot analysis and immunoprecipitation were done as previously described [48]. For Myc and β-actin, the protein bands were detected by LI-COR imaging system (LI-COR Bioscience, Lincoln, NE, USA). Chemiluminescence was used to detect all other antibodies. The CK2 kinase activity assay was performed as described previously [49]. Briefly, the cell lysates were collected in hypo-osmotic buffer after treatment with PBS (control) or IL-6 (20 ng/ml) for 20 min, and the cell lysates (5 µg), synthetic peptide CK2 substrate (RRRADDSDDDDD; 0.1 mM), and γ-^32^P-ATP in the assay dilution buffer were incubated for 10 min at 30°C. The phosphorylated substrate was then separated on P81 phosphocellulose paper and quantified with a scintillation counter.

### Boyd chamber migration assay and wound-healing assay

The transwell migration assay was performed over a 20-h period using 24-well Boyden chamber system plates from BD Biosciences (San Jose, CA, USA). Cells that had migrated across the filters were counted using Image J software. The wound-healing assay was done as described previously [50].

### Statistical analysis

The Student *t*-test was used for all statistical analyses, and a *P* value less than 0.05 was considered statistically significant.

## Supporting Information

Figure S1 **Expression of the receptor for IL-6 (IL-6R) in SCCHN cells.** The levels of *IL-6R* mRNA expression were normalized to the expression levels of the housekeeping gene *GAPDH* and were expressed as the mean fold change from basal ± s.e.m. Because there is no detectable mRNA expression in cell lines HOK16B and Nom 9, all expression levels were normalized to that of SQCCY1. All experiments were done in triplicate for each cell line. (* *P*<0.05)
(TIF)

Figure S2 **Not only IL-6 but also EGF and VEGF-C can upregulate Twist expression in SCCHN cells.** Twist protein expression was induced shortly after IL-6 (20 ng/ml), EGF (20 ng/ml) or VEGF-C (50 ng/ml) in OSC-19 SCCHN cells. Twist protein expression in cell lysates after indicated treatment was analyzed by western blot; β-actin was used as a loading control.
(TIF)

Table S1 **Levels of secreted IL-6 in SCCHN cell lines.** Levels of secreted IL-6 in the supernatants of cultured SCCHN cells were determined by using a human IL-6 ELISA kit after cells were cultured in 2% complete medium for 48 h. Data are expressed as pg/million cells ± s.e.m. All of the SCCHN lines secreted increased levels of IL-6 relative to the immortalized human keratinocye cell line (NOM 9).
(EPS)
