# Supplementary figures and images for: IL-6 Stabilizes Twist and Enhances Tumor Cell Motility in Head and Neck Cancer Cells through Activation of Casein Kinase 2
Source: PLoS One. 2011 Apr 29;6(4):e19412. doi: 10.1371/journal.pone.0019412 (PMC3084854; doi:10.1371/journal.pone.0019412)

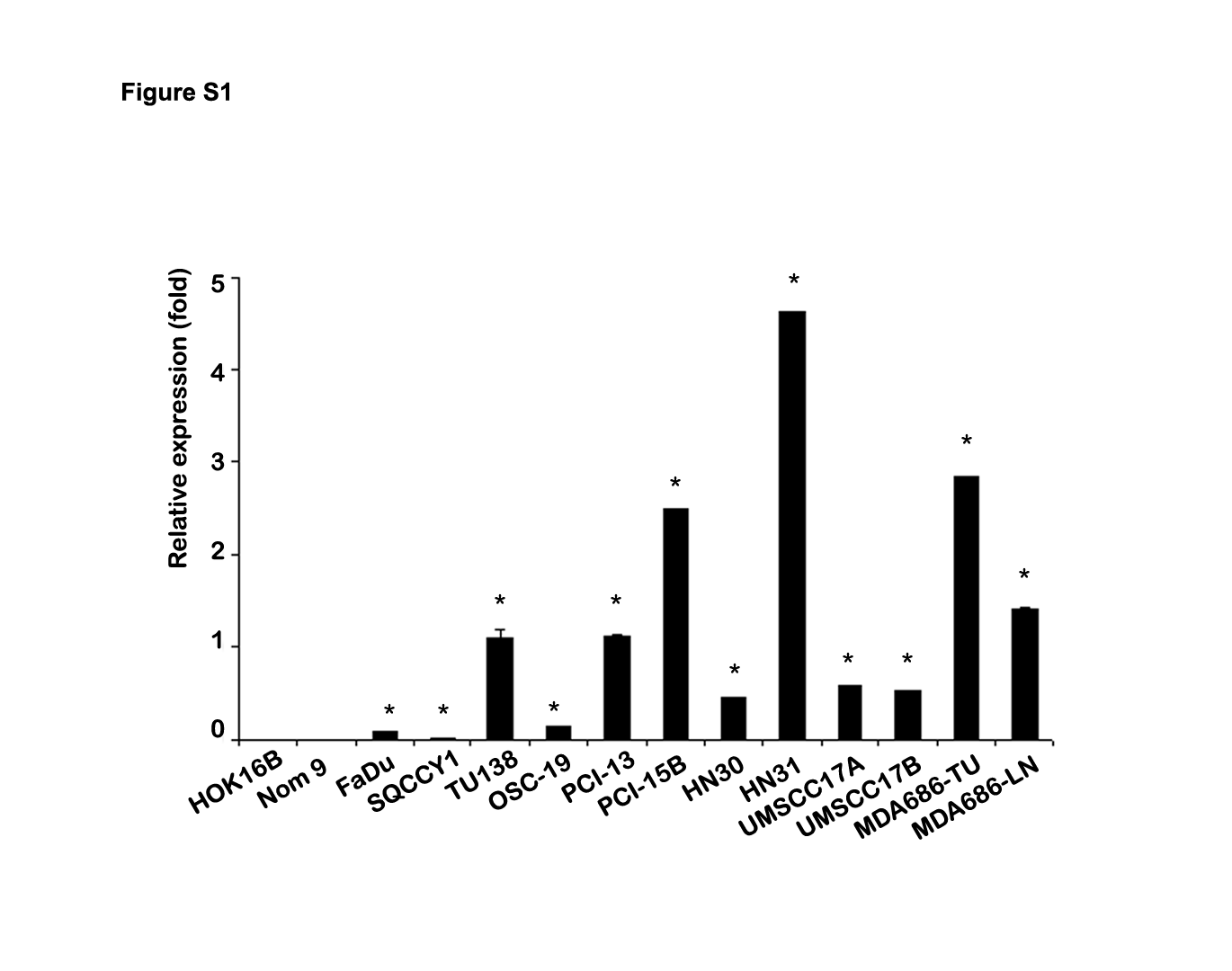

Supplement: Figure S1 — Expression of the receptor for IL-6 (IL-6R) in SCCHN cells. The levels of IL-6R mRNA expression were normalized to the expression levels of the housekeeping gene GAPDH and were expressed as the mean fold change from basal ± s.e.m. Because there is no detectable mRNA expression in cell lines HOK16B and Nom 9, all expression levels were normalized to that of SQCCY1. All experiments were done in triplicate for each cell line. (* P<0.05) (TIF) [file pone.0019412.s001.tif]

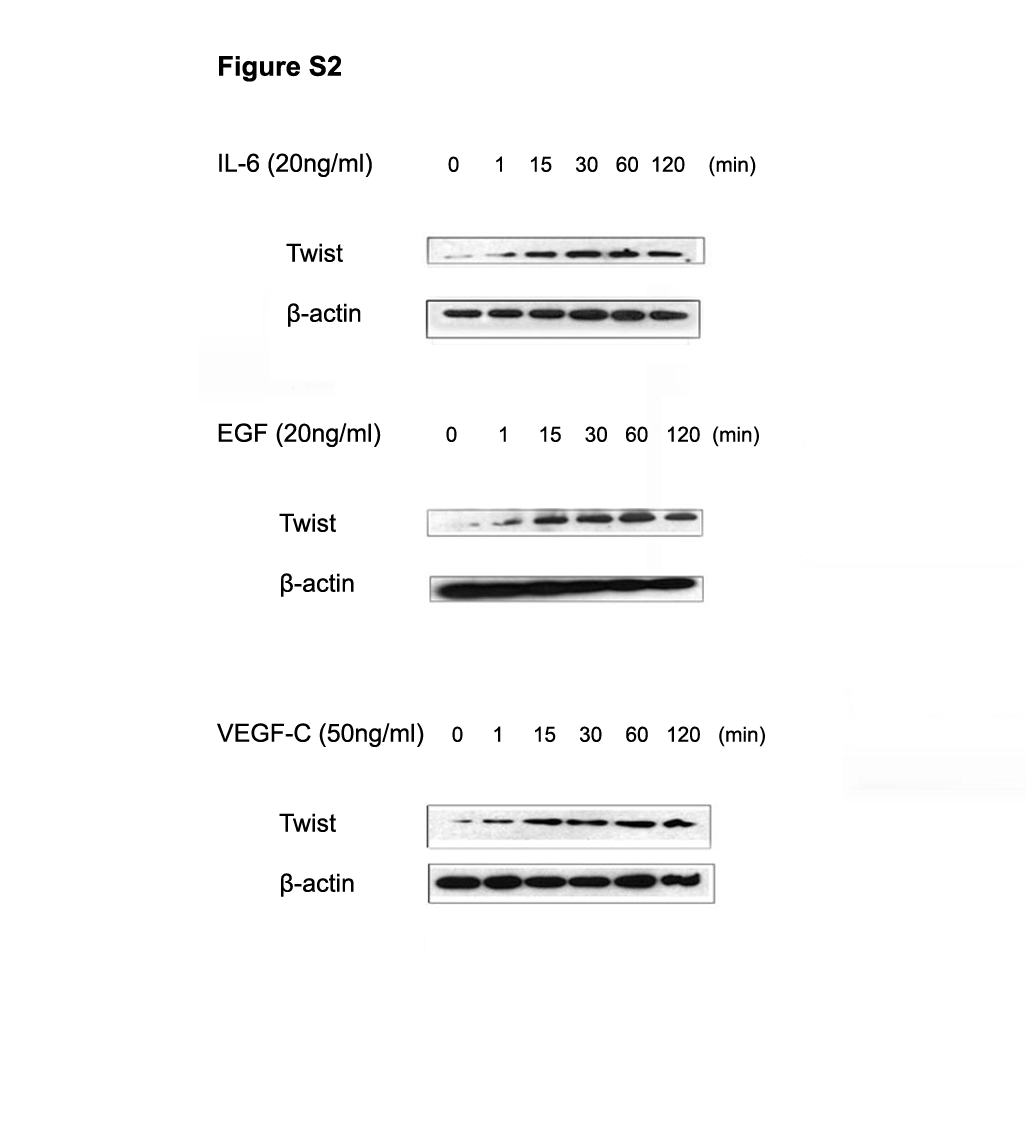

Supplement: Figure S2 — Not only IL-6 but also EGF and VEGF-C can upregulate Twist expression in SCCHN cells. Twist protein expression was induced shortly after IL-6 (20 ng/ml), EGF (20 ng/ml) or VEGF-C (50 ng/ml) in OSC-19 SCCHN cells. Twist protein expression in cell lysates after indicated treatment was analyzed by western blot; β-actin was used as a loading control. (TIF) [file pone.0019412.s002.tif]
